# Perception of the Online Learning Environment of Nursing Students in Slovenia: Validation of the DREEM Questionnaire

**DOI:** 10.3390/healthcare9080998

**Published:** 2021-08-05

**Authors:** Lucija Gosak, Nino Fijačko, Carolina Chabrera, Esther Cabrera, Gregor Štiglic

**Affiliations:** 1 Faculty of Health Sciences, University of Maribor, 2000 Maribor, Slovenia; nino.fijacko@um.si (N.F.); gregor.stiglic@um.si (G.Š.); 2 Research Group in Attention to Chronicity and Innovation in Health (GRACIS), TecnoCampus, Universitat Pompeu Fabra, 08002 Barcelona, Spain; cchabrera@tecnocampus.cat (C.C.); ecabrera@tecnocampus.cat (E.C.); 3 Faculty of Electrical Engineering and Computer Science, University of Maribor, 2000 Maribor, Slovenia; 4 Usher Institute, University of Edinburgh, Edinburgh EH8 9YL, UK

**Keywords:** education, learning environment, nursing student, transcultural adaptation, psychometric properties, health care

## Abstract

At the time of the outbreak of the coronavirus pandemic, several measures were in place to limit the spread of the virus, such as lockdown and restriction of social contacts. Many colleges thus had to shift their education from personal to online form overnight. The educational environment itself has a significant influence on students’ learning outcomes, knowledge, and satisfaction. This study aims to validate the tool for assessing the educational environment in the Slovenian nursing student population. To assess the educational environment, we used the DREEM tool distributed among nursing students using an online platform. First, we translated the survey questionnaire from English into Slovenian using the reverse translation technique. We also validated the DREEM survey questionnaire. We performed psychometric testing and content validation. I-CVI and S-CVI are at an acceptable level. A high degree of internal consistency was present, as Cronbach’s alpha was 0.951. The questionnaire was completed by 174 participants, of whom 30 were men and 143 were women. One person did not define gender. The mean age of students was 21.1 years (SD = 3.96). The mean DREEM score was 122.2. The mean grade of student perception of learning was 58.54%, student perception of teachers was 65.68%, student academic self-perception was 61.88%, student perception of the atmosphere was 60.63%, and social self-perception of students was 58.93%. Although coronavirus has affected the educational process, students still perceive the educational environment as positive. Nevertheless, there is still room for improvement in all assessed areas.

## 1. Introduction

Due to the coronavirus pandemic (COVID-19), which was reported in Wuhan, China [1,2,3,4] and soon after, the first major outbreak in Europe spread rapidly to Slovenia [5,6]. Governments issued directives on social isolation and living at home, so colleges and universities around the world were closed [7]. COVID-19 has forced education systems around the world to find alternatives to personal teaching [8]. Online distance learning platforms are the only available way of learning and teaching during unprecedented events such as the outbreak of COVID-19 [9,10,11]. However, it is important to distinguish between online distance education and distance learning in an emergency as a temporary solution. Online education provides students with flexibility and choice [12]. This involves implementing education using information and communication technology [13] and represents an easily accessible teaching method [14]. 

Online learning promotes student-centered learning, in which case courses are easy to manage [15], resulting in better knowledge and self-efficacy for some students [16]. It increases performance, encourages critical thinking, and improves writing skills for most students [17]. Through the accelerated use of online learning, educators and carers need to consider the pedagogical and practical challenges posed by the integration of online learning [18]. Negative aspects highlighted are a lack of appropriate infrastructure for some students, less effective communication and interaction, inability to implement practical applications, lack of socialization, lack of motivation, less objective exams, and the possibility of deteriorating health [19]. 

Despite growing evidence that online learning is just as effective as traditional learning tools, there is very little evidence of what works, when, and how online learning improves teaching and learning [20]. Therefore, in this study, we decided to evaluate the online learning environment of students using the Dundee Ready Education Environment Measure (DREEM) tool [21,22,23]. Any learning environment that meets students’ internal and external needs is likely to lead to better and more promising learning outcomes [24]. Achieving an optimal educational environment must meet the expectations of students regarding the school atmosphere, teaching, teachers, students, school staff, educational equipment, and the physical environment [25]. A good learning environment for students in clinical practice depends on the structure of student admission, the pedagogical atmosphere, and the participation of those involved [26]. The educational environment has an impact on students’ learning outcomes, preparation for practice, and student satisfaction [27]. Also, the perception of the learning environment is related to well-being and stress in students [28].

The main goal of the research is a validation of the questionnaire focusing on the assessment and perception of nursing students about the online learning environment. The goal is also to test psychometrically the DREEM tool [22,23]. The validation of the DREEM tool is performed within the Erasmus+ project Digital Toolbox for Innovation and Nursing Education (I-BOX), which aims to develop material for teaching nursing students and nurses. Based on the obtained results, we will also assess where the greatest deviations occur in the assessment of the learning environment and thus encourage the improvement of the learning environment for students.

## 2. Materials and Methods

### 2.1. Study Design

We used quantitative research methodology [29,30,31]. Data for assessing the educational environment by undergraduate and postgraduate nursing students were collected using an online questionnaire between November 2020 and January 2021. The survey questionnaire was previously translated into Slovenian language and validated in the Slovenian environment for the first time.

### 2.2. Assessment Tool

To assess the online educational environment, we used the DREEM tool [22,23]. DREEM is a validated tool for assessing the educational environment in health care professions worldwide [32]. In addition to being used to diagnose deficiencies in the current educational environment, DREEM is also used to compare different groups, monitor the same group over time, and assess factors influencing the educational environment [33,34]. The DREEM tool includes five subscales: students’ perception of learning (SPL); students’ perception of teachers (SPT); students’ academic self-perception (SAP); students’ perception of the atmosphere (SPA) and students’ social self-perception (SSP). The maximum score is 200 [35]. The use of the questionnaire was previously authorized by the authors [22,23]. The survey questionnaire was translated from English into Slovenian and then back to the original language [36]: Independently by two researchers, the survey questionnaire was translated from English into Slovenian. Both researchers had the necessary knowledge of English, andragogy, and nursing. Thus, we obtained two versions of the translation, which we merged into one in the next step, based on consultation between experts. If disagreement was present, a third researcher was involved. In the last step, two experts with the necessary knowledge of English translated a joint version of the Slovenian questionnaire into English. Thus, we obtained two forms of reverse translation and subsequently merged them into a common form [29,30].

Questionnaires were distributed using an online survey platform ENKA from which the results were then downloaded and analysed using IBM SPSS Statistics 27.

#### 2.2.1. Validation of Assessment Tool

We assessed the validity of the content and the validity of the construct in the survey questionnaire and performed confirmatory factor analysis [37,38]. To determine the content validity, we included experts who have the necessary knowledge in the field [29,30,37,39]. Based on the recommendations where six to ten experts are required [40], we included six experts who work as nursing teachers. The questions in the questionnaire were rated on a four-point scale from 1 to 4, where 1 represents statements that are not relevant; 2, deficient/poorly understood statements; 3, partially understandable/partially relevant statements; and 4, entirely understandable/completely relevant claims [41]. To assess the content validity of the questionnaire, we calculated the content validity of individual claims (I-CVI) and content validity of the whole questionnaire (S-CVI) [41,42,43,44,45,46]. For the internal reliability analysis, we calculated Cronbach’s α, which presents us with a measure of internal reliability between several items [47]. Cronbach’s alpha coefficients and interpreted the values as follows: ≥0.90, excellent; 0.80–0.89, good; 0.70–0.79, acceptable; 0.60–0.69, questionable; 0.50–0.59, poor; and <0.50, unacceptable [48]. Correlations between items are an essential element in the analysis of the items representing a specific concept. Correlations between items examine the extent to which ratings of one item are related to ratings of all other scale items [49,50,51].

I-CVI represents the quotient between the number of experts who rated each question with a grade of 3 or 4 and between the number of all experts, which in our case was six [42,44,45,46,52]. The probability of agreement was calculated using the formula Pc = [N!/A! (N-A)!] 0,5N where N represents the number of evaluators, and A represents the number of consents [42,44,45,46,52,53]. We used the following formula to calculate the kappa determination of the compliance agreement: k = (I-CVI − Pc)/(1 − Pc). I-CVI represents item content validity index, and Pc represents the probability of chance agreement [42,44,45,46,52]. The S-CVI represents the proportion of questions rated by two experts with a score of 3 or 4 [39,42,52].

#### 2.2.2. Perception of the Learning Environment

The DREEM tool includes 50 items, 41 positive and nine negatives, related to learning perception (12 items), teacher perception (11 items), academic self-perception (eight items), atmospheric perception (12 items), and social self-perception (seven items). Each item is rated on a five-point Likert scale (from 1—strongly disagree to 5—strongly agree), where reverse-coding is used for nine statements [22,32]. Questions 4, 8, 9, 17, 25, 35, 39, 48, and 50 are reverse-coded [22,32,54]. The highest score indicates an ideal educational environment [22,32]. The categorization of the sub-scale for all items is as follows: lower than 50 represents a very poor level, range 51–75 is defined as a “plenty of problems” category, range 76–150 represents more positive than negative category, and higher than 150 represents an excellent score [35]. When analysing an individual item, it is necessary to pay attention to those with a mean score lower than 2. There are also possible improvements in the measured assumptions with a mean score between 2 and 3 [55,56,57].

### 2.3. Ethics of Research

Before the research, we obtained ethical permission from the institutional ethical commission (No. 038/2020/2176-02/504). The authors of the questionnaire were asked for permission to use and translate it. Individuals who submitted responses to the online questionnaire also agreed to participate in the survey [22,23]. As part of the research, we sent students an invitation to participate in the research by e-mail. The online questionnaire also informed the participants about the purpose and goals of the research. Participants had the opportunity to refuse to participate in the anonymous survey. The survey was conducted from November 2020 until January 2021. We also informed them that we would use the results exclusively for research. In doing so, we will not disclose information from which the individuals involved could be identified. The risks and burdens of research are minimal.

## 3. Results

Of the 298 invited participants, 174 participants completed the questionnaire (response rate: 58.4%). Of these, 17% (*n* = 30) were men and 83% (*n* = 143) were women (one person did not specify their gender). The average age of the participants was 21.1 years (SD = 3.96). The youngest person was 18 years old, and the oldest was 46 years old. Other basic characteristics of the students involved are shown in Table 1.

### 3.1. DREEM Tool Validation Results

The DREEM questionnaire was backtranslated from English into Slovenian by two experts. The content validity and reliability of the DREEM tool questionnaire in the Slovenian environment to assess the perception of the learning space in nursing students are presented below.

#### 3.1.1. Content Validity of the Questionnaire

Table 2 presents the I-CVI, Pc, and k coefficient calculations for all questions in the DREEM tool. I-CVI for all questions in the Slovenian version of the questionnaire is acceptable. The I-CVI for all questions except question 20 was 1.000. The I-CVI for question twenty, “The teaching is well focused,” was 0.833. The probability of agreement on all questions is 0.016, and on the twentieth question, 0.094. Kappa on the determination of the agreement on adequacy for all questions is 1. For the twentieth question, it is 0.816.

The evaluation of two experts was included in the S-CVI assessment. None of them rated the question with a score of 1 or a score of 2 with a final S-CVI of 1.000 and is acceptable for the Slovenian environment (Table 3).

#### 3.1.2. Reliability of the Questionnaire

Supplementary Materials presents the correlations between the items in each scale in the DREEM tool questionnaire. Item correlations ranged between −0.038 and 0.620.

Cronbach’s alpha was 0.951, which indicates a high level of internal consistency. Table 4 represents the values of Cronbach’s alpha with specific items deleted. Removing any question other than question 17, “Cheating is a problem in this school,” and question 25, “The teaching over-emphasizes factual learning,” would reduce the value of Cronbach’s alpha. Corrected item-total correlation for question 17 was 0.186, and 0.192 for question 25.

**Figure 1:**
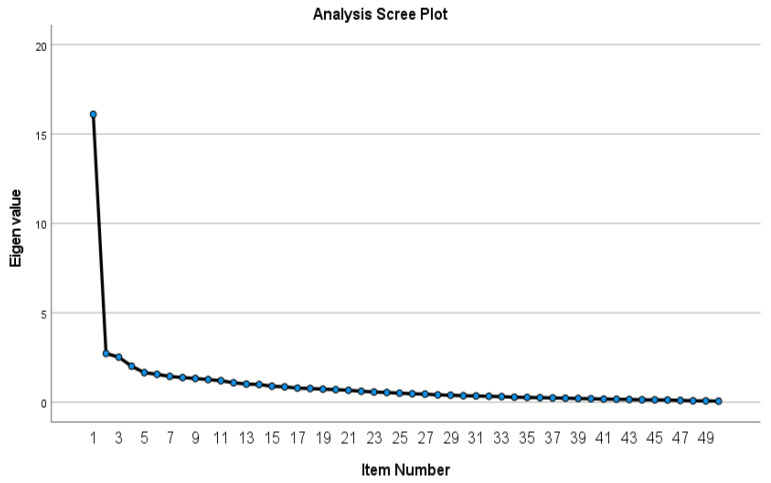
Analysis Scree Plot.

Figure 1 presents a graph for screen analysis. The graph shows the eigenvalue scree plot for 50 instrument elements and points at one factor.

### 3.2. Results of Perception of the Learning Environment

Online teaching was perceived more positively than negatively. The mean assessment of student perception of learning is 28.1/48, student perception of teachers is 28.9/44, student academic self-perception is 19.8/32, student perception of the atmosphere is 29.1/48, and social self-perception of students is 16.5/28 (Table 5). All individual subscales are statistically related (*p* < 0.001).

**Figure 2:**
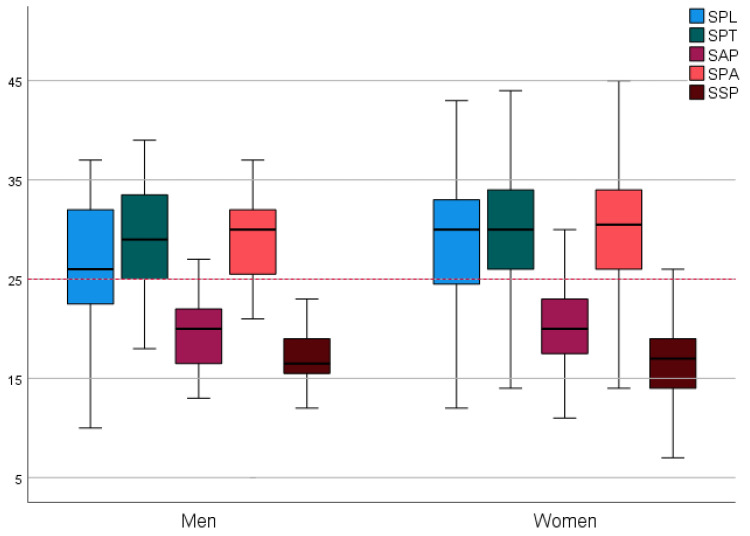
Gender comparison in subscales.

Based on the Shapiro–Wilk test for women and the Kolmogorov–Smirnov test for men, we found that the individual values of the scales in students were unevenly distributed according to gender. Based on the Mann–Whitney U test, we identified a statistically significant relationship between the assessment of student perception of learning by gender (U = 1346,500; *p* = 0.024). The mean SPL score for men was 24.9/48 (SD = 8.82). For women, this mean score was 28.9/48 (SD = 7.27). There is no statistically significant difference by gender between the other subscales. Nevertheless, in all subscales, the scores were higher for women than for men: subscale SPT (29.3 vs. 28.1), subscale SAP (20.0 vs. 19.0), subscale SPA (29.4 vs. 28.6), and subscale SSP (16.4 vs. 16.6) (Figure 2).

To show the relationship between age and individual subscales, we performed a Pearson correlation test. The age of students is statistically significantly related to the SAP subscale score (r = 0.212; *p* = 0.007) and the SPA subscale score (r = 9.213; 0.007). Based on the Kruskal–Wallis test, we found that the study program attended by students affects the SAP score. The mean grade of SAP students attending the undergraduate first degree study program nursing care is 19.7/32 (SD = 5.05), the score of students attending the postgraduate second degree study program nursing care is 25.67/32 (1.53), and the score of students who attend a postgraduate third degree study program nursing care is 26/32 (SD = 8.49). 

The mean assessment of student perception of learning is 28.1/48, which means a more positive perception. Problematic assumptions with a mean grade of ≤2 in the SPL subscale are “I am encouraged to participate in class,” which has an average grade of 1.8 (SD = 0.83), and “The teaching over-emphasizes factual learning,” which has a mean grade of 1.3 (SD = 0.68) (Table 6); 69.2% of men (*n* = 18) and 64% of women (*n* = 80) agree that teachers being encouraging to participate. Table S1 in Supplementary Materials present the links between SPL items.

The mean score of student perception of teachers is 28.9/44, which means that it is moving in the right direction. The item “The teachers are authoritarian” received the lowest mean value of 1.9 (SD = 0.98) (Table 7); 39.4% of respondents (*n* = 62) do not agree with this statement, and 25.5% (*n* = 48) neither agree nor disagree with this statement. Table S2 in Supplementary Materials present the links between SPT items. 

The mean score of students’ academic self-perception is 19.8/32, representing that feelings are more on the positive side. None of the items in the SAP subscale received a lower mean score than 2 (Table 8). With the highest mean score, the item “I have learned a lot about empathy in my profession” stands out, with a mean score of 3.1 (SD = 0.65). A total of 89.9% of respondents (*n* = 134) agree that they learned a lot about empathy in the profession during their studies in the current year. Table S3 in Supplementary Materials present the links between SAP items.

A score of students’ perceptions of the atmosphere is 29.1/48, meaning that the atmosphere is more positive than negative. The lowest score was given to the statement “This school is well timetabled” and was 1.5 (SD = 1.10) (Table 9); 51.3% of respondents (*n* = 81) disagree that the schedule is well planned, 25.9% (*n* = 41) neither agree nor disagree with the statement. Table S4 in Supplementary Materials present the links between SPA items.

The mean score of students’ social self-perception is 16.5/28, meaning that social perception is not too bad (Table 10). The item “There is a good support system for students who get stressed” and the item “I am too tired to enjoy this course” get a lower score of 2, more specifically 1.8 (SD = 1.06) and 1.7 (SD = 0.97). 43.1% of the surveyed (*n* = 69) students are too tired to participate in the lectures. Table S4 in Supplementary Materials present the links between SPL items.

Supplementary Materials represents the inter-item correlations of the subscale.

## 4. Discussion

To the best of the authors ‘knowledge, this is the first study to assess students’ perceptions of the educational environment in Slovenia. We wanted to obtain information to assess the learning environment of nursing students. Our study was conducted during the COVID-19 pandemic, when colleges were forced to move their education online. Thus, despite the challenges of social distancing, isolation, and quarantine measures [58], they continued to provide education for nurses [59]. 

The assessment of the learning environment in the nursing student participants of this study is more positive than negative, as in many studies where this tool was used [15,35,54,60,61,62,63,64,65,66,67,68]. So far, only one study has been conducted that provides researchers with insight into the differences between personal and online teaching. In the United Kingdom, researchers conducted a national cross-sectional study to assess the learning environment during online teaching. They found that the assessment of the learning environment was lower than in live teaching [21].

We wanted to assess if there are differences between individual scales according to gender. In our study, differences were detected only in the assessment of learning perception (SPL), where women had a higher score than men (28.9 for women vs. 24.9 for men, *p* = 0.024). No statistically significant differences were detected in other subscales. The overall score is also higher for women (124.3; SD = 29.04) compared with men (116.1; SD = 32.1). Similar results were also obtained in another study where researchers found higher scores in women than in men [62]. This means that women have a better perception of the educational environment. Studies detect gender differences in study habits, which in turn affect student outcomes [69]. Also, female students are more willing to participate and work in a team than male students [70]. There are also differences in the acceptance of e-learning between men and women [71]. In contrast to our study, however, Fooladi found that perceptions of the learning environment are lower in women among vulnerable groups [72].

There is no statistically significant difference between years of enrolment in our study. The highest DREEM score is detected in the first year of study, where the mean grade is 124.15 (SD = 31.89). Other research finds that perception of the learning environment differs according to student performance, and also a difference between individual years of study [73]. Shrestha, et al., also note that the learning environment assessment is highest among students in the first year of study [74]. 

Of particular concern is that most students disagree with the claim that the schedule is well planned. Only 20.7% of respondents (*n* = 36) rate schedules as well-planned. This can also be related to the observation that 40.2% of students (*n* = 70) are often too tired to participate in lectures. Students are primarily concerned with time management in distance learning [75,76]. It is important to reduce the academic burden on students and help students develop time management skills, which significantly contributes to their success [77,78]. Stress and overload in nursing students can lead to burnout, anxiety, and depression [79]. 

Nebhinani, et al. point out that there is a great need to plan and implement various stress management programs [80]. Only 23.5% of respondents (*n* = 41) in our study agree that a good support system is in place in the presence of stress. Like our study, students in eastern Nepal perceived that they do not have a good support system during times of stress [74]. Numerous studies have found increased stress in students due to an outbreak of coronavirus disease [10,81,82,83], so support in this area is particularly important at this time. Stress connected with distance learning for students mainly leads to a lack of concentration, motivation, and technical difficulties [84].

56.3% of students (*n* = 98) believe that teachers focus too much on teaching based on data memorization, and 36.2% of students (*n* = 63) believe that teachers are too authoritative in their work. Nevertheless, most students (*n* = 117; 67.2%) believe that teaching is sufficiently focused on developing competencies related to their profession. 

Health science students will receive such a good education, but its effectiveness must be rigorously and regularly evaluated [85]. Therefore, it is of the utmost importance that such research is continued, and the rate of improvement is assessed. Only in this way can we achieve the best possible learning environment for students.

### Limitations

There is a possibility of bias due to low response to the survey questionnaire. The reason for this might be in the fact that questionnaires were sent to the students in an online form, which usually results in low response rates. The study also took place within one faculty and cannot be generalized on a wider scale. Also, the limitation is that the assessment of the educational environment was carried out only during online teaching and cannot be compared with the evaluation of the learning environment during the traditional implementation of the learning process. Another limitation is that the online survey was conducted only from November 2020 to January 2021 and not in other study periods.

## 5. Conclusions

Nursing students generally rate their learning environment more positively than negatively, but there is still room for improvement in all categories. Greater emphasis is needed on the organization and timing of lessons to achieve better concentration of students in classes and reduce their level of stress. Educational organizations are also recommended to set up a good support system for students. The need to change the approach by teachers and their role was also perceived. With an authoritative approach and too much emphasis on factual learning, we negatively affect the student’s motivation and willingness to work. Teachers can improve this through appropriate pedagogical and andragogic education. 

It is important that learning organizations and teachers also focus on providing a suitable and appropriate learning environment for students during distance learning. This is the only way they can contribute to positive learning outcomes and gain student experience. However, this presents a unique challenge, as the teacher has no contact with students when teaching online. 

In the future, we plan to conduct a longitudinal study to observe the impact and variation of different factors in assessment of the learning environment over time.

## Figures and Tables

**Table 1:** Sample characteristics.

**Gender**	**N (%)**
Men	30 (17.2%)
Female	143 (82.2%)
Missing	1 (0.6%)
**Age**	**M (SD)**
	21.1 (3.96)
**Study program**	**N (%)**
Undergraduate 1st degree study programme Nursing Care	167 (96%)
Postgraduate 2nd degree study programme Nursing Care	3 (1.7%)
Postgraduate 3rd degree study programme Nursing Care	2 (1.1%)
Missing	2 (1.1%)
**Study year**	**N (%)**
1st year	86 (49.4%)
2nd year	59 (33.9%)
3rd year	23 (13.2%)
Senior	5 (2.9%)
Missing	1 (0.6%)

N = sample size; % = percent.

**Table 2:** Content validity of the DREEM tool.

No.	Question(s)	N	A	I-CVI	Pc	k	Interpretation
1	I am encouraged to participate in class.	6	6	1.000	0.016	1.000	Appropriate
2	The teachers are knowledgeable.	6	6	1.000	0.016	1.000	Appropriate
3	There is a good support system for students who get stressed.	6	6	1.000	0.016	1.000	Appropriate
4	I am too tired to enjoy this course.	6	6	1.000	0.016	1.000	Appropriate
5	Learning strategies which worked for me before continue to work for me now.	6	6	1.000	0.016	1.000	Appropriate
6	The teachers are patient with patients.	6	6	1.000	0.016	1.000	Appropriate
7	The teaching is often stimulating.	6	6	1.000	0.016	1.000	Appropriate
8	The teachers ridicule the students.	6	6	1.000	0.016	1.000	Appropriate
9	The teachers are authoritarian.	6	6	1.000	0.016	1.000	Appropriate
10	I am confident about my passing this year.	6	6	1.000	0.016	1.000	Appropriate
11	The atmosphere is relaxed during the ward teaching.	6	6	1.000	0.016	1.000	Appropriate
12	This school is well timetabled.	6	6	1.000	0.016	1.000	Appropriate
13	The teaching is student-centred.	6	6	1.000	0.016	1.000	Appropriate
14	I am rarely bored on this course.	6	6	1.000	0.016	1.000	Appropriate
15	I have good friends in this school.	6	6	1.000	0.016	1.000	Appropriate
16	The teaching is sufficiently concerned to develop my competence.	6	6	1.000	0.016	1.000	Appropriate
17	Cheating is a problem in this school.	6	6	1.000	0.016	1.000	Appropriate
18	The teachers have good communications skills with patients.	6	6	1.000	0.016	1.000	Appropriate
19	My social life is good.	6	6	1.000	0.016	1.000	Appropriate
20	The teaching is well focused.	6	5	0.833	0.094	0.816	Appropriate
21	I am feel am being well prepared for my profession.	6	6	1.000	0.016	1.000	Appropriate
22	The teaching is sufficiently concerned to develop my confidence.	6	6	1.000	0.016	1.000	Appropriate
23	The atmosphere is relaxed during lectures.	6	6	1.000	0.016	1.000	Appropriate
24	The teaching time is put to good use.	6	6	1.000	0.016	1.000	Appropriate
25	The teaching over-emphasizes factual learning.	6	6	1.000	0.016	1.000	Appropriate
26	Last year work has been a good preparation for this year’s work.	6	6	1.000	0.016	1.000	Appropriate
27	I am able to memorize all I need.	6	6	1.000	0.016	1.000	Appropriate
28	I seldom feel lonely.	6	6	1.000	0.016	1.000	Appropriate
29	The teachers are good at providing feedback to students.	6	6	1.000	0.016	1.000	Appropriate
30	There are opportunities for me to develop interpersonal skills.	6	6	1.000	0.016	1.000	Appropriate
31	I have learned a lot about empathy in my profession.	6	6	1.000	0.016	1.000	Appropriate
32	The teachers provide constructive criticism here.	6	6	1.000	0.016	1.000	Appropriate
33	I feel comfortable in class socially.	6	6	1.000	0.016	1.000	Appropriate
34	The atmosphere is relaxed during seminars/tutorials.	6	6	1.000	0.016	1.000	Appropriate
35	I find the experience disappointing.	6	6	1.000	0.016	1.000	Appropriate
36	I am able to concentrate well.	6	6	1.000	0.016	1.000	Appropriate
37	The teachers give clear examples.	6	6	1.000	0.016	1.000	Appropriate
38	I am clear about the learning objectives of the course.	6	6	1.000	0.016	1.000	Appropriate
39	The teachers get angry in class.	6	6	1.000	0.016	1.000	Appropriate
40	The teachers are well prepared for their class.	6	6	1.000	0.016	1.000	Appropriate
41	My problem-solving skills are being well developed here.	6	6	1.000	0.016	1.000	Appropriate
42	The enjoyment outweighs the stress of studying medicine.	6	6	1.000	0.016	1.000	Appropriate
43	The atmosphere motivates me as a learner.	6	6	1.000	0.016	1.000	Appropriate
44	The teaching encourages me to be an active learner.	6	6	1.000	0.016	1.000	Appropriate
45	Much of what I have to learn seems relevant to a career in medicine.	6	6	1.000	0.016	1.000	Appropriate
46	My accommodation is pleasant.	6	6	1.000	0.016	1.000	Appropriate
47	Long-term learning is emphasized over short-term.	6	6	1.000	0.016	1.000	Appropriate
48	The teaching is too teacher-centred.	6	6	1.000	0.016	1.000	Appropriate
49	I feel able to ask the questions I want.	6	6	1.000	0.016	1.000	Appropriate
50	The students irritate the teachers.	6	6	1.000	0.016	1.000	Appropriate

No. = Number of question; N = sample size; A = number of agreements; I-CVI = item content validity index; Pc = probability of chance agreement; k = kappa designating agreement on relevance.

**Table 3:** Scale content validity of the DREEM tool.

	Expert Ratter No. 1	Expert Ratter No. 2	Total
Items rated 1 or 2	0	0	0
Items rated 3 or 4	50	50	100
Items rated 3	11	2	13
Items rated 4	39	48	87
S-CVI	50/50 = 1.000		

S-CVI = scale content validity.

**Table 4:** Item-total statistics.

No.	Scale Mean if Item Deleted	Scale Variance if Item Deleted	Corrected Item-Total Correlation	Cronbach’s Alpha if Item Deleted
1	127.8932	555.567	0.501	0.950
2	126.6893	555.765	0.507	0.950
3	127.9029	541.912	0.652	0.949
4	127.9223	552.896	0.468	0.951
5	127.0194	560.882	0.384	0.951
6	126.7864	557.052	0.443	0.951
7	127.2039	545.399	0.689	0.949
8	126.5049	552.743	0.571	0.950
9	127.6019	554.673	0.435	0.951
10	126.7087	561.875	0.340	0.951
11	127.1456	550.283	0.557	0.950
12	128.0777	545.072	0.581	0.950
13	127.2136	549.189	0.596	0.950
14	127.5728	553.678	0.478	0.951
15	126.5340	563.898	0.339	0.951
16	126.7961	554.791	0.597	0.950
17	126.9806	567.862	0.186	0.952
18	126.6893	558.785	0.552	0.950
19	126.8738	557.053	0.423	0.951
20	126.9223	553.386	0.688	0.950
21	126.9709	558.715	0.419	0.951
22	127.1262	547.111	0.664	0.949
23	126.8932	553.430	0.683	0.950
24	126.7767	556.352	0.678	0.950
25	128.3204	567.573	0.192	0.952
26	126.8932	562.018	0.364	0.951
27	127.4660	549.800	0.557	0.950
28	127.1553	556.780	0.397	0.951
29	126.8835	550.006	0.668	0.950
30	126.8641	556.060	0.614	0.950
31	126.5631	562.621	0.450	0.951
32	126.9515	556.341	0.570	0.950
33	126.5728	558.208	0.549	0.950
34	126.6699	556.164	0.607	0.950
35	127.0583	545.820	0.715	0.949
36	127.1748	561.714	0.376	0.951
37	126.8932	549.077	0.700	0.949
38	126.9806	553.078	0.510	0.950
39	126.8252	555.655	0.513	0.950
40	126.8058	549.609	0.693	0.949
41	126.9612	548.979	0.780	0.949
42	127.4757	544.075	0.651	0.949
43	127.2718	545.769	0.663	0.949
44	127.2621	546.215	0.682	0.949
45	126.9806	560.706	0.361	0.951
46	126.3786	569.198	0.232	0.951
47	127.0000	544.843	0.672	0.949
48	127.1748	564.479	0.299	0.951
49	126.7184	556.322	0.520	0.950
50	126.7670	561.024	0.385	0.951

**Table 5:** Mean score of DREEM tool.

Subscale	Items	Total Score	Mean Score (SD)	Maximum Score	Minimum Score	Interpretation
SPL	12	48	28.1 (7.92)	47	3	A more positive approach (25–36)
SPT	11	44	28.9 (7.31)	44	5	Moving in the right direction (23–33)
SAP	8	32	19.8 (5.26)	32	4	Feeling more on the positive side (17–24)
SPA	12	48	29.1 (8.35)	48	3	A more positive atmosphere (25–36)
SSP	7	28	16.5 (3.93)	28	2	Not too bad (15–21)
Total	50	200	122.2 (30.66)	196	20	More positive than negative (101–150)

SPL = Students perception of learning; SPT = Students perception of teachers; SAP = Students academic self-perception; SPA = Students perceptions of atmosphere; SSP = Students social self-perceptions; SD = standard deviation.

**Table 6:** Subscale SPL.

No.	Question(s)	N	M (SD)
1	I am encouraged to participate in class.	160	1.8 (0.83)
7	The teaching is often stimulating.	160	2.5 (0.88)
13	The teaching is student-centred.	158	2.5 (0.94)
16	The teaching is sufficiently concerned to develop my competence.	160	2.8 (0.78)
20	The teaching is well focused.	159	2.7 (0.70)
22	The teaching is sufficiently concerned to develop my confidence.	150	2.6 (0.89)
24	The teaching time is put to good use.	152	2.9 (0.61)
25	The teaching over-emphasizes factual learning.	151	1.3 (0.68)
38	I am clear about the learning objectives of the course.	150	2.7 (0.86)
44	The teaching encourages me to be an active learner.	147	2.4 (0.91)
47	Long-term learning is emphasized over short-term.	147	2.7 (0.95)
48	The teaching is too teacher-centred.	148	2.5 (0.79)

No. = Number of question; N = sample size; M = mean; SD = standard deviation.

**Table 7:** Subscale SPT.

No.	Question(s)	N	M (SD)
2	The teachers are knowledgeable.	160	3.1 (0.84)
6	The teachers are patient with patients.	148	2.9 (0.88)
8	The teachers ridicule the students.	159	3.1 (0.83)
9	The teachers are authoritarian.	174	1.9 (0.98)
18	The teachers have good communications skills with patients.	144	3.0 (0.69)
29	The teachers are good at providing feedback to students.	150	2.8 (0.78)
32	The teachers provide constructive criticism here.	143	2.7 (0.72)
37	The teachers give clear examples.	151	2.8 (0.78)
39	The teachers get angry in class.	151	2.8 (0.84)
40	The teachers are well prepared for their class.	151	2.9 (0.79)
50	The students irritate the teachers.	147	2.9 (0.83)

No. = Number of question; N = sample size; M = mean; SD = standard deviation.

**Table 8:** Subscale SAP.

No.	Question(s)	N	M (SD)
5	Learning strategies which worked for me before continue to work for me now.	157	2.6 (0.81)
10	I am confident about my passing this year.	161	2.9 (0.84)
21	I am feel am being well prepared for my profession.	149	2.7 (0.83)
26	Last year work has been a good preparation for this year’s work.	113	2.7 (0.81)
27	I am able to memorize all I need.	152	2.2 (0.95)
31	I have learned a lot about empathy in my profession.	174	3.1 (0.65)
41	My problem-solving skills are being well developed here.	146	2.7 (0.74)
45	Much of what I have to learn seems relevant to a career in medicine.	147	2.7 (0.83)

No. = Number of question; N = sample size; M = mean; SD = standard deviation.

**Table 9:** Subscale SPA.

No.	Question(s)	N	M (SD)
11	The atmosphere is relaxed during the ward teaching.	144	2.5 (0.95)
12	This school is well timetabled.	159	1.5 (1.10)
17	Cheating is a problem in this school.	160	2.7 (0.87)
23	The atmosphere is relaxed during lectures.	151	2.8 (0.69)
30	There are opportunities for me to develop interpersonal skills.	152	2.7 (0.74)
33	I feel comfortable in class socially.	151	3.0 (0.69)
34	The atmosphere is relaxed during seminars/tutorials.	150	3.0 (0.66)
35	I find the experience disappointing.	151	2.7 (0.86)
36	I am able to concentrate well.	151	2.5 (0.79)
42	The enjoyment outweighs the stress of studying medicine.	147	2.1 (0.98)
43	The atmosphere motivates me as a learner.	147	2.4 (0.96)
49	I feel able to ask the questions I want.	147	3.0 (0.79)

No. = Number of question; N = sample size; M = mean; SD = standard deviation.

**Table 10:** Subscale SSP.

No.	Question(s)	N	M (SD)
3	There is a good support system for students who get stressed.	161	1.8 (1.06)
4	I am too tired to enjoy this course.	161	1.7 (0.97)
14	I am rarely bored on this course.	159	2.1 (0.95)
15	I have good friends in this school.	159	3.0 (0.81)
19	My social life is good.	158	2.8 (0.96)
28	I seldom feel lonely.	151	2.5 (0.97)
46	My accommodation is pleasant.	146	3.2 (0.67)

No. = Number of question; N = sample size; M = mean; SD = standard deviation.

## Data Availability

Data is currently not available for sharing, due to the further data collection process. Contact the first author for more information.

## Supplementary Materials

The following are available online at https://www.mdpi.com/article/10.3390/healthcare9080998/s1, Inter-item correlations of the subscale.
