# Supplementary material for: Perception of the Online Learning Environment of Nursing Students in Slovenia: Validation of the DREEM Questionnaire
Source: Healthcare (Basel). 2021 Aug 5;9(8):998. doi: 10.3390/healthcare9080998 (PMC8391444; doi:10.3390/healthcare9080998)
Supplement: Supplementary file 1 [file healthcare-09-00998-s001.zip › healthcare-1326542-supplementary.pdf]

healthcare

MDPI

Supplementary Material A: Inter-item correlations of the Subscale

Table S1: Inter-item correlations of the Subscale Students' Perception of Learning (SPL).

Correlation between SPL items												
	**Q1**	**Q7**	**Q13**	**Q16**	**Q20**	**Q22**	**Q24**	**Q25**	**Q38**	**Q44**	**Q47**	**Q48**
**Q1**	1.000	0.515	0.395	0.221	0.324	0.344	0.423	0.057	0.306	0.350	0.414	0.132
**Q7**	0.515	1.000	0.591	0.360	0.573	0.405	0.486	0.140	0.384	0.440	0.482	0.174
**Q13**	0.395	0.591	1.000	0.396	0.563	0.523	0.418	0.274	0.322	0.409	0.510	0.107
**Q16**	0.221	0.360	0.396	1.000	0.535	0.403	0.459	0.165	0.241	0.450	0.479	0.175
**Q20**	0.324	0.573	0.563	0.535	1.000	0.514	0.437	0.059	0.470	0.505	0.585	0.098
**Q22**	0.344	0.405	0.523	0.403	0.514	1.000	0.450	0.100	0.304	0.475	0.545	0.067
**Q24**	0.423	0.486	0.418	0.459	0.437	0.450	1.000	0.162	0.321	0.518	0.456	0.220
**Q25**	0.057	0.140	0.274	0.165	0.059	0.100	0.162	1.000	0.029	0.185	0.137	0.240
**Q38**	0.306	0.384	0.322	0.241	0.470	0.304	0.321	0.029	1.000	0.278	0.366	0.206
**Q44**	0.350	0.440	0.409	0.450	0.505	0.475	0.518	0.185	0.278	1.000	0.547	0.131
**Q47**	0.414	0.482	0.510	0.479	0.585	0.545	0.456	0.137	0.366	0.547	1.000	0.157
**Q48**	0.132	0.174	0.107	0.175	0.098	0.067	0.220	0.240	0.206	0.131	0.157	1.000

Q = question.

Table S2: Inter-item correlations of the Subscale Students' Perception of Teachers (SPT).

Correlation between SPT items											
	**Q2**	**Q6**	**Q8**	**Q9**	**Q18**	**Q29**	**Q32**	**Q37**	**Q39**	**Q40**	**Q50**
**Q2**	1.000	0.382	0.167	0.000	0.418	0.382	0.205	0.495	0.279	0.558	0.165
**Q6**	0.382	1.000	0.160	0.106	0.483	0.285	0.241	0.398	0.228	0.485	0.195
**Q8**	0.167	0.160	1.000	0.379	0.286	0.391	0.299	0.379	0.499	0.350	0.404
**Q9**	0.000	0.106	0.379	1.000	0.082	0.197	0.151	0.170	0.230	0.242	0.193
**Q18**	0.418	0.483	0.286	0.082	1.000	0.493	0.414	0.486	0.268	0.557	0.257
**Q29**	0.382	0.285	0.391	0.197	0.493	1.000	0.516	0.598	0.438	0.543	0.295
**Q32**	0.205	0.241	0.299	0.151	0.414	0.516	1.000	0.457	0.182	0.420	0.216
**Q37**	0.495	0.398	0.379	0.170	0.486	0.598	0.457	1.000	0.390	0.620	0.271
**Q39**	0.279	0.228	0.499	0.230	0.268	0.438	0.182	0.390	1.000	0.382	0.490
**Q40**	0.558	0.485	0.350	0.242	0.557	0.543	0.420	0.620	0.382	1.000	0.291
**Q50**	0.165	0.195	0.404	0.193	0.257	0.295	0.216	0.271	0.490	0.291	1.000

Q = question.

Table S3: Inter-item correlations of the Subscale Students' Academic-Self Perception (SAP).

Correlation between SAP items								
	**Q5**	**Q10**	**Q21**	**Q26**	**Q27**	**Q31**	**Q41**	**Q45**
**Q5**	1.000	0.106	0.248	0.290	0.173	0.300	0.290	0.142
**Q10**	0.106	1.000	0.351	0.082	0.389	0.142	0.341	0.021
**Q21**	0.248	0.351	1.000	0.115	0.170	0.267	0.346	0.002
**Q26**	0.290	0.082	0.115	1.000	0.246	0.289	0.343	0.098
**Q27**	0.173	0.389	0.170	0.246	1.000	0.106	0.494	0.290
**Q31**	0.300	0.142	0.267	0.289	0.106	1.000	0.402	0.210
**Q41**	0.290	0.341	0.346	0.343	0.494	0.402	1.000	0.306
**Q45**	0.142	0.021	0.002	0.098	0.290	0.210	0.306	1.000

Q = question.

healthcare MDPI

Table S4: Inter-item correlations of the Subscale Students' Perception of Atmosphere (SPA).

Correlation between SPA items												
	**Q11**	**Q12**	**Q17**	**Q23**	**Q30**	**Q33**	**Q34**	**Q35**	**Q36**	**Q42**	**Q43**	**Q49**
**Q11**	1.000	0.369	-0.029	0.469	0.309	0.345	0.487	0.402	0.169	0.384	0.376	0.235
**Q12**	0.369	1.000	-0.009	0.350	0.304	0.143	0.325	0.473	0.278	0.321	0.417	0.128
**Q17**	-0.029	-0.009	1.000	0.117	0.164	0.150	0.244	0.139	0.037	-0.038	0.081	0.359
**Q23**	0.469	0.350	0.117	1.000	0.401	0.385	0.498	0.416	0.305	0.415	0.528	0.460
**Q30**	0.309	0.304	0.164	0.401	1.000	0.342	0.301	0.439	0.358	0.302	0.477	0.385
**Q33**	0.345	0.143	0.150	0.385	0.342	1.000	0.566	0.323	0.197	0.349	0.363	0.280
**Q34**	0.487	0.325	0.244	0.498	0.301	0.566	1.000	0.384	0.127	0.396	0.304	0.432
**Q35**	0.402	0.473	0.139	0.416	0.439	0.323	0.384	1.000	0.356	0.423	0.532	0.339
**Q36**	0.169	0.278	0.037	0.305	0.358	0.197	0.127	0.356	1.000	0.235	0.378	0.137
**Q42**	0.384	0.321	-0.038	0.415	0.302	0.349	0.396	0.423	0.235	1.000	0.563	0.330
**Q43**	0.376	0.417	0.081	0.528	0.477	0.363	0.304	0.532	0.378	0.563	1.000	0.290
**Q49**	0.235	0.128	0.359	0.460	0.385	0.280	0.432	0.339	0.137	0.330	0.290	1.000

Q = question.

Table S5: Inter-item correlations of the Subscale Students' Social-Self Perception (SSP).

Correlation between SSP items							
	**Q3**	**Q4**	**Q14**	**Q15**	**Q19**	**Q28**	**Q46**
**Q3**	1.000	0.269	0.345	0.185	0.233	0.157	0.041
**Q4**	0.269	1.000	0.226	0.069	0.116	0.138	0.171
**Q14**	0.345	0.226	1.000	0.226	0.019	0.155	0.060
**Q15**	0.185	0.069	0.226	1.000	0.120	0.200	0.114
**Q19**	0.233	0.116	0.019	0.120	1.000	0.322	0.109
**Q28**	0.157	0.138	0.155	0.200	0.322	1.000	0.116
**Q46**	0.041	0.171	0.060	0.114	0.109	0.116	1.000

Q = question.
